# A Genome-wide Study of “Non-3UTR” Polyadenylation Sites in *Arabidopsis thaliana*

**DOI:** 10.1038/srep28060

**Published:** 2016-06-15

**Authors:** Cheng Guo, Matthew Spinelli, Man Liu, Qingshun Q. Li, Chun Liang

**Affiliations:** 1 Department of Biology, Miami University, Oxford, OH 45056, USA; 2 Key Laboratory of the Ministry of Education for Costal and Wetland Ecosystems, College of the Environment and Ecology, Xiamen University, Xiamen, Fujian 361102, China; 3 Graduate College of Biomedical Sciences, Western University of Health Sciences, Pomona, CA 91766, USA

## Abstract

Alternative polyadenylation has been recognized as a key contributor of gene expression regulation by generating different transcript isoforms with altered 3′ ends. Although polyadenylation is well known for marking the end of a 3′ UTR, an increasing number of studies have reported previously less-addressed polyadenylation events located in other parts of genes in many eukaryotic organisms. These other locations include 5′ UTRs, introns and coding sequences (termed herein as non-3UTR), as well as antisense and intergenic polyadenlation. Focusing on the non-3UTR polyadenylation sites (**n3PAS**s), we detected and characterized more than 11000 **n3PAS** clusters in the Arabidopsis genome using poly(A)-tag sequencing data (PAT-Seq). Further analyses suggested that the occurrence of these **n3PASs** were positively correlated with certain characteristics of their respective host genes, including the presence of spliced, diminutive or diverse beginning of 5′ UTRs, number of introns and whether introns have extreme lengths. The interaction of the host genes with surrounding genetic elements, like a convergently overlapped gene and associated transposable element, may contribute to the generation of a **n3PAS** as well. Collectively, these results provide a better understanding of **n3PAS**s, and offer some new insights of the underlying mechanisms for non-3UTR polyadenylation and its regulation in plants.

Polyadenylation plays an essential role during the post-transcriptional maturation process of precursor mRNA (pre-mRNA) in eukaryotes. A typical polyadenylation process consists of a cleavage of pre-mRNA at the polyadenylation site (or poly(A) site) and a subsequent synthesis of a poly(A) tail at the 3′ end onto the pre-mRNA. The process of utilizing different poly(A) sites along individual pre-mRNAs, also known as alternative polyadenylation (APA), has been increasingly recognized as a key contributor of gene expression regulation^1^. More than 80% of human genes and over 75% of Arabidopsis genes possess APA sites, resulting in various mRNA isoforms differing in their 3′ ends or coding sequences^2^,^3^. It is known that polyadenylation is conducted by the polyadenylation machinery complex that exerts its function by recognizing *cis*-element sequences embedded in the pre-mRNA^1^,^4^. The usage of one poly(A) site over the other is likely due to the relative “strength” of the polyadenylation signals being perceived by the poly(A) machinery, as well as the availability of the poly(A) sites and transacting protein factors^5^,^6^. Therefore, both the presence of polyadenylation machinery and the appropriate sequence context around a poly(A) site are essential for polyadenylation. Besides these two major regulators, other factors such as the process of transcription initiation, stem-loop motifs in 3′ untranslated regions (UTRs), epigenetics modification and splicing factors were also found to be involved in the process of polyadenylation^7^,^8^,^9^,^10^,^11^. Given the complexity, the current understanding of APA regulation is still insufficient for us to elucidate the precise selection and expressional preference among APA sites within the same genes. The mechanisms governing both global and gene-specific APA are only starting to be deciphered.

With the advances of high-throughput sequencing techniques and the development of bioinformatics methodologies and tools in the past decade, three previously less-addressed polyadenylation events, *i.e.*, antisense, intergenic, and non-3UTR polyadenylation sites (located in 5′-UTR, intron and coding sequences, collectively termed **n3PAS**s), have been found in almost all systems, raising interesting questions about their authenticity, biological relevance and underlying molecular mechanisms^12^,^13^,^14^,^15^,^16^,^17^,^18^,^19^,^20^,^21^. An antisense poly(A) transcript is termed so as its direction runs opposite to its host gene. Typically in Arabidopsis, about one third of poly(A) site clusters (PACs) are anti-sense, impacting either 3213^12^, 7600^13^ or 12090^14^ genes by different studies. Although the nature of antisense poly(A) sites remains unknown, many individual antisense PACs have been determined functional with a major involvement in transcriptional silencing^15^,^16^. Regarding intergenic poly(A) sites, whole genome tiling arrays and next generation sequencing data suggest the existence of polyadenylated transcripts in 19~23% of the *Arabidopsis* intergenic regions, where no genic units are annotated in both strands^3^. A recent study has suggested many of these intergenic poly(A) sites are evidence of novel noncoding or protein-coding genes in currently annotated intergenic regions^17^. Lastly, in contrast to the majority (83%) of sense PACs that occur within the 3′ UTR^3^, the remaining sense PACs are defined as **n3PAS**s accordingly. They are further classified into 5′ UTR, CDS exon and CDS intron located PACs by their relative locations in terms of their longest annotated transcript isoforms of genes. Distinct from 3′ UTR APA that only varies in the 3′ UTR, **n3PAS** could change the coding sequence with a truncated length and even differently spliced isoforms. Although documented by independent studies for years^18^,^19^,^20^,^21^, the stability and functionality of **n3PAS**-based transcripts are still controversial. Moreover, Sandberg and his colleagues suggested that coding region and 3′ UTR poly(A) sites were regulated differently in human T-cells, indicating **n3PAS**s and 3′ UTR APAs were undergoing partially different regulatory mechanisms^22^.

Using the genome browser of Arabidopsis from the Arabidopsis Information Resource (TAIR, https://www.arabidopsis.org/), a preliminary visual inspection of these **n3PAS**s, by aligning the custom track that contains positional and expression information of poly(A) sites to the reference, suggested that **n3PAS**s were usually positively correlated with some atypical gene features. To characterize these **n3PAS**s and possibly further explore the underlying mechanisms of their formation, a comprehensive polyadenylation dataset from Arabidopsis was collected utilizing next generation sequencing data^12^,^23^. The subsequent genome-wide bioinformatics analyses confirmed that the occurrences of these **n3PAS**s were strongly correlated with certain characteristics of their host genes. Surrounding genes and transposable elements (TEs) may also contribute to the generation of **n3PAS**s. Collectively, as the first systematic research in characterizing **n3PAS**s in eukaryotes, our work provides a comprehensive study of non-3UTR polyadenylation, and unveils another layer of regulatory mechanism for alternative polyadenylation selection in plants.

## Methods

### PAT Preparation and poly(A) sites identification

Raw RNA-Seq data from four independent studies were collected. To note, three of them were based on the poly(A) tag sequencing (PAT-Seq, by the Illumina platform) method^24^, and the last one was based on the direct RNA sequencing (DRS, by the Helicos platform) method^25^. For simplicity and consistency, sequence reads from both PAT-Seq and DRS sequencing, which had been used to define poly(A) sites unambiguously, were called PAT reads. For the PAT-Seq based samples, libraries were prepared from different tissues of wild-type plants (Col-0: CS6000), including leaf (named PAT_L, accession number: SRA048565)^23^, root (named PAT_R, accession number: SRS1295579) and unopened flower (named PAT_F, accession number: SRS1295578). The detailed procedures for growing wild-type plants, isolating RNA, preparing and sequencing PATs had been reported elsewhere^23^,^24^. In particular, PAT_R and PAT_F were new datasets generated by us, which have not been analyzed and reported previously. The DRS dataset (named DRS_L, accession number: ERP001018) was generated from total RNA isolated from young leaves of wild-type plants (Col-0)^12^. Although the polyadenylation annotation for PAT_L and DRS_L was provided through their original publications^12^,^24^, all datasets were reprocessed individually using the same custom-built bioinformatics pipeline in our study to maintain consistency. The bioinformatics procedure for extracting poly(A) sites from PAT-Seq data followed the previously described protocol^26^, with a slight change that GSNAP^27^ was used instead of Helisphere^28^ as the alignment tool for mapping PAT reads to the reference genome. After mapping aforementioned datasets, each successfully aligned PAT read defined an individual poly(A) site. To avoid the internal priming, the genomic sequence of −10 to +5 *nt* surrounding the poly(A) site (position = 0) was retrieved firstly by the *get-genome* program of GSNAP, and then a customized PERL script was used to scan through this poly(A) site flanking region. If this region has eight or more continuous As, the putative poly(A) site was considered as an internal priming case, which was filtered out from downstream analyses. We retained high-quality poly(A) sites (*i.e.*, those supported with ≥3 PAT reads) and discarded low-quality poly(A) sites (*i.e.*, those supported with <3 PAT reads). Given the fact that poly(A) site microheterogeneity was ubiquitous in eukaryotes including Arabidopsis, poly(A) sites in the vicinity were clustered into PACs in our study as previously described^29^,^30^. Within each PAC cluster, the poly(A) site possessing the most mapped PAT reads was selected as the representative site for that PAC.

### Annotation of poly(A) site clusters

The relative genic locations of PACs were critical to distinguish different groups or categories of PACs in our analysis. To study the relative locations of PACs, genes and transposable elements, we built a MySQL database holding the directional and positional information of these genic elements using Python. The database was populated in part by parsing the TAIR10 GFF files and relevant transposable elements annotations (ftp://ftp.arabidopsis.org/). Protein coding genes were exclusively selected, and their longest transcripts were only used as the reference if individual genes have multiple transcript isoforms annotated. Sequences from chloroplast or mitochondria were ignored. The terminal position of the 3′ UTR of each transcript was further extended for 120 *nt* to accommodate potential missing annotations of 3′ UTR poly(A) sites as suggested previously^3^. Accordingly, sequences between any of these extended transcript isoforms were defined as intergenic regions. Once the genomic coordinate information of PACs was obtained, the classification of all PACs was conducted using PERL scripts by mapping PAC locations to our extended annotations.

### Tools/methods for characterization of poly(A) site clusters

Different groups of PACs were characterized by quantifying the relative base composition on a nucleotide-by-nucleotide basis, extending from 100 *nt* upstream (−100) to 100 *nt* downstream (+100) of each PAC site (position = 0). The sequences were extracted using the *get-genome* program in GMAP/GSNAP package (version 2015-07-23)^31^. The sequence profiles were plotted using CLC genomic workbench (http://www.clcbio.com). To estimate the authenticity of PACs from high-throughput sequencing data among different PAC categories or groups, PASS (Poly(A) Site Sleuth)^32^ was used to calculate the prediction scores. As required by the tool, sequences of the PAC site flanking regions (−300 *nt* to +100 *nt*) were collected from different PAC categories based on their relative locations in individual genes. The positional score was the average score of the typical positions of all flanking sequences in a given category. To examine if a given PAC category is involved in possible biological functions, gene functional annotation was performed using the DAVID Bioinformatics Resources^33^ (https://david.ncifcrf.gov/).

### Statistical analysis and visualization

To evaluate the statistical significance of whether **n3PAS**s are correlated with specific gene characteristics and to compare the lengths of introns with and without **n3PAS**s, T-test and Chi-square test were performed in R (http://www.R-project.org). The P-value of 0.05 was used to define statistical significance. Plots were generated using SAS (www.SAS.com) and Excel. Snapshots of **n3PAS** relative positions to genes were obtained from TAIR genome browser (https://www.arabidopsis.org/).

## Results

### Characterization of poly(A) site distribution in four datasets

**Figure 1:**
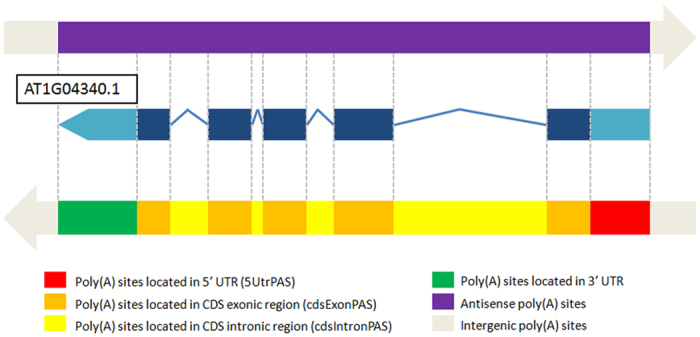
A diagram describing the definitions of different categories or groups of poly(A) site clusters (PACs). The top and bottom panel represent the direction of poly(A) sites to the right and left respectively. Different colors in the top and bottom panels represent different PAC categories based on where the poly(A) site cluster is located in the corresponding genetic region. Specifically, PACs located in the 5′ UTRs (5UtrPASs), CDS exonic regions (cdsExonPASs) and CDS intronic regions (cdsIntronPASs) are classified as non-3UTR poly(A) sites (**n3PASs**), in contrast to those located in 3′ UTRs (3UtrPASs).

Many APA sites were identified in different regions of genes in Arabidopsis and other eukaryotes. However, how these genic regions can influence the selection of the APA sites was not clearly understood, particularly for those APAs located in the regions other than 3′UTRs. To this end, we summarized those **n3PAS**s and asked if there was any relationship between them and the characteristics of their corresponding genic regions. Six major PAC categories or groups were defined and determined as shown in Fig. 1. As shown in Table 1, among the four datasets, the data with greater sequencing depths showed more PACs. Also, while the PAC distribution was slightly different among four datasets, constant fractions of intergenic and antisense PACs were found in all datasets. The majority of PATs from the entire sense PATs population were in the 3′ UTR (3UtrPASs), with an average of 97.8% for PAT-Seq based datasets and 97.1% for the DRS based dataset. The high proportion of 3′ UTR PACs were also in accordance with the previous studies^3^,^12^. To simplify the analyses, all PAT reads from the three PAT-Seq based datasets were pooled together to form a new dataset (PAT_LFR), while the DRS dataset was not pooled, because PAT-Seq based datasets requires cDNA formation using RNA templates in sequencing while DRS does not. PACs were subsequently clustered and summarized, as shown by the PAT_LFR dataset in Table 1. In total, 36984 sense PACs were detected in the protein coding gene regions, consisting of 11782 **n3PAS** PACs and 25202 3′ UTR PACs. To note, there were 7330 CDS exon PACs in PAT_LFR, while only 612 were in DRS_L. Such difference between PAT_LFR and DRS_L was also consistent with the previous studies^12^,^34^.

### Characterization and examples of n3PASs located in different genic regions

Different regions of the pre-mRNA may have different characteristics in the transcription process, thus analysis of **n3PAS**s of different genic regions may delineate the characteristics of their host genes.

#### Poly(A) sites located in 5′ UTR (5UtrPASs)

Many 5UtrPASs are likely mis-annotated by the previous identification algorithms^3^, because these sites were probably 3′ UTR poly(A) sites from the upstream genes when two convergent genes were nearby. Using 400 *nt* as the cutoff distance, a custom PERL script was written to exclude these potentially mis-annotated 5UtrPASs. A total of 146 suspicious 5UtrPASs (composed of 113 exon located and 33 intron located 5UtrPASs) from the PAT_LFR dataset were identified, which were later classified as 3′ UTR poly(A) sites (3UtrPASs) from their upstream genes. The remaining 598 5UtrPASs (with 345664 PAT reads) were from 481 individual genes. The enriched function for those genes were studied in DAVID bioinformatics resources, which suggested that the most enriched functional category was associated with response to abiotic stimuli, osmotic stresses and salt stresses. Specifically, the gene AT1G20920 had the highest number (7) of 5′ UTR PACs (Supplementary Figure 1A). As illustrated in TAIR10, it contained two RNA transcript isoforms, and their 5′ UTRs were spliced and had inconsistent locations. Moreover, AT1G20920 was known as a plant stress regulation related factor^35^. Although 5UtrPASs were rarely documented^36^, evidence for their existence can also be spotted in the current TAIR10 annotation, especially when viewed in light of our result of 5UtrPASs from PAT_LFR, such as the case for gene AT1G07590 and AT1G07600 (Supplementary Figure 2). Interestingly, instead of annotating this event as an **n3PAS**, it was annotated as a new embedded gene (AT1G07600) in TAIR10, even though it was likely a 5UtrPAS originated from gene AT1G07590 with a high expression level. In addition, a few other cases associated with 5′ UTR poly(A) events in PAT_LFR suggested that 5UtrPASs may confuse the current annotation pipeline used in TAIR10 by generating error-prone annotations. For instance, gene cluster of AT5G50010, AT5G50011 and AT5G50012 seemed mis-annotated (Supplementary Figure 3).

#### Poly(A) sites located in the CDS intronic regions (cdsIntronPASs)

There were 3708 cdsIntronPASs (tallied from 350389 PATs) in the PAT_LFR dataset. Interestingly, these cdsIntronPASs were likely located in longer introns. The mean intron length with and without cdsIntronPASs in our study was 398.5 and 163.3 *nt* respectively (P-value < 2.2e-16, Supplementary Figure 4). In addition, the mean intron length with cdsIntronPASs increased to 460.9 *nt* when only considering high-quality PACs with a higher coverage (i.e., PAT reads>10). Also, cdsIntronPASs were normally located in the last CDS intron right before the 3′ UTR, especially when cdsIntronPASs have a relative higher expression. The enriched function for genes containing these cdsIntronPASs suggested the most enriched functional category was associated with defense response, apoptosis and cell death. The gene with the greatest number (9) of cdsIntronPASs was AT3G05165 (Supplementary Figure 1B). Regarding this gene, although the end of its longest transcript (AT3G05165.5) was not confirmed by the PAT_LFR dataset, one of the shorter transcripts was confirmed by the most abundant PAC that belonged to this gene. Notably, the most abundant cdsIntronPASs (with 2196 PATs) were located in the last CDS intron right before the 3′ UTR when using the longest transcript as the reference. In addition, AT3G05165 contained two long introns (both were around 1,000 *nt* in length) and 96% (2235/2324) of PATs were located in those two introns.

#### Poly(A) sites located in the CDS exonic regions (cdsExonPASs)

In PAT_LFR, 7330 cdsExonPASs (compiled from 225716 PATs) were detected. As mentioned, most cdsExonPASs present in PAT-Seq were not supported by DRS. The result of enriched function analysis for those genes suggested no significantly enriched function either. The gene with the most cdsExonPASs was AT5G40450 (Supplementary Figure 1C), which contained 33 cdsExonPASs. In contrast, no cdsExonPASs were detected for the DRS dataset within AT5G40450. Multiple reasons could lead to this discrepancy, including the differences in plant material sampled and sequencing methods applied.

### Poly(A) signal profile comparison among different PAC categories or groups

**Figure 2:**
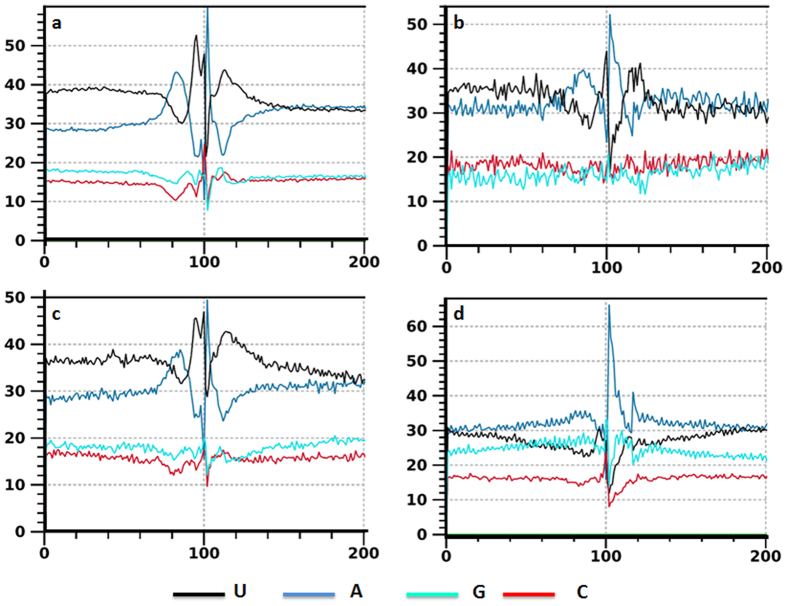
Position-by-position nucleotide composition plots around polyadenylation sites within different genomic regions. The positions of 100 at x-axis indicate the poly(A) site positions. (**a**) PACs that map to 3′ UTRs. (**b**) PACs that map to 5′ UTRs. (**c**) PACs that map to CDS intronic regions. (**d**) PACs that map to CDS exonic regions.

**Figure 3:**
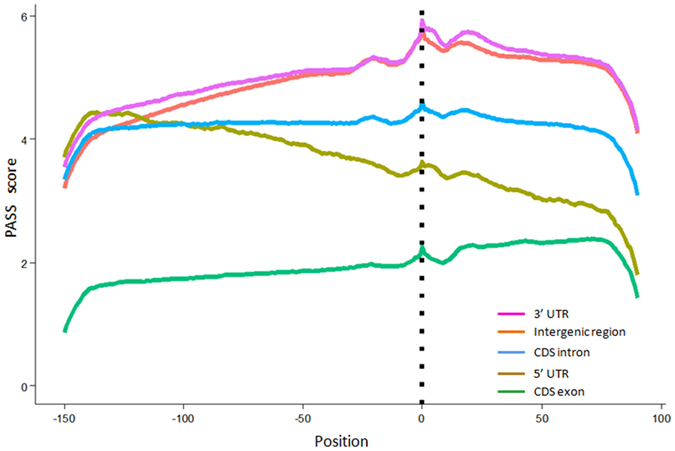
Averaged PASS scores of the flanking sequences of polyadenylation sites (−152~+100 *nt*) within different genomic regions.

To understand why different genic region localized PACs were chosen, and how they were recognized, nucleotide composition profile analysis was performed as it reflects the overall differences in poly(A) signals among these different PAC categories or groups^17^,^37^. As illustrated in Fig. 2, the profiles of position-by-position nucleotide composition of 3′ UTR, 5′ UTR and CDS intronic PACs were similar. These poly(A) sites were flanked by regions with distinctive nucleotide composition preferences, displaying a dominant and alternating pattern of A/U composition over C/G. These profiles generally reflected genuine cleavage sites with the poly(A) signals recognized by polyadenylation machinery as demonstrated previously^2^,^3^. However, the profile of PACs located in CDS exons was very different from those of the other three genic locations (Fig. 2), having a higher A/G composition and no alternating pattern of A/U sequences was observed before the cleavage site. This is in line with a previous analysis^17^. Additionally, PASS was adopted to evaluate the authenticity of PACs. A higher PASS score indicates a higher probability of being a real poly(A) site^32^. The output scores for each position of the flanking PAC sequences of each PAC category/group were averaged and plotted in Fig. 3. We observed similar patterns at poly(A) site locations with a sharp peak among all sense PAC groups, including intergenic poly(A) sites, suggesting that these positions provide the most favorable condition for polyadenylation. In accordance with a previous study, intergenic PACs shared very similar sequence characteristics as 3′ UTR PACs^17^. However, the average score of all **n3PAS**s displayed a lower value than both 3′ UTR and intergenic PACs, even though the sharp peak patterns were evident at the poly(A) sites in all groups, indicating that the poly(A) sites were the most favorable cleavage sites for both **n3PAS**s and 3′ UTR poly(A) sites, but meanwhile **n3PAS**s have an overall lower opportunity to embrace polyadenylation.

### Characteristics of genes associated with n3PAS

Only some genes have **n3PAS**s, hence characterizing the features of **n3PAS** hosted genes would be helpful to understand the mechanisms generating **n3PAS**s. A few genic features of host genes were summarized from preliminary inspection of individual genes by TAIR10 genome browser.

#### Genes containing an “atypical” 5′ UTR

**Figure 4:**
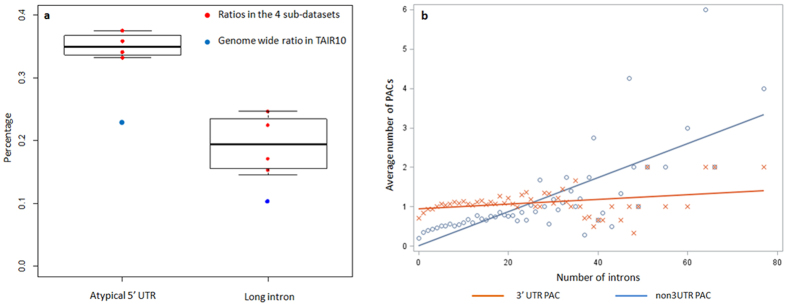
Genome-wide characterization of host genes that contain non-3UTR poly(A) sites. (**a**) Comparing with the reference TAIR10 annotation, both the ratios of atypical 5′ UTR events and long intron events for the genes that contain **n3PAS**s in four sub-datasets are significantly different. (**b**) The comparison of the average abundance between non-3UTR and 3′ UTR PACs in the host genes with different intron numbers. The X-axis presents the number of introns in the group of host genes. The Y-axis presents the average number of PACs, which is equal to the number of total PACs divided by the total number of genes in that group.

Several “atypical” 5′ UTR features were defined and summarized during the initial visual inspection, namely “spliced 5′ UTRs”, “ambiguous 5′ UTRs” (inconsistent 5′ UTRs between isoforms) and “extreme short 5′ UTRs” (Supplementary Figure 5). Specifically, if any gene transcript was annotated to contain an intron in the 5′ UTR, then the gene was labeled as a “spliced 5′ UTR” gene. If a gene possessed two or more annotated transcript isoforms, and its 5′ UTR annotation has different starting or ending positions among its isoforms, then this gene was labeled as an “ambiguous 5′ UTR” gene. Further, if the 5′ UTR was very short (≤10 *nt*), then it was labeled as an “extreme short 5′ UTR” gene. Conflicts were resolved when classifying a 5′ UTR by assigning precedence as follows: spliced > ambiguous > extreme short 5′ UTR. In TAIR10, among 27206 protein coding genes, 6087 (22.4%) of them possess an “atypical” 5′ UTR feature (4177 spliced 5′ UTRs, 1422 ambiguous 5′ UTRs and 488 extreme short 5′ UTRs). In contrast, among all these 11782 **n3PAS**s, 3872 (32.9%) of their host genes contain an “atypical” 5′ UTR feature (2758 spliced 5′ UTRs, 961 ambiguous 5′ UTRs and 153 extreme short 5′ UTRs). To assess the statistical significance of “atypical” 5′ UTRs in **n3PAS**s contained genes, the frequency of “atypical” 5′ UTRs for host genes with **n3PAS**s were obtained from all four aforementioned datasets (PAT_L, PAT_F, PAT_R and DRS_L). Compared to the whole genome level (TAIR10), the frequency of “atypical” 5′ UTR events in genes containing **n3PAS**s was significantly higher (P-value = 0.0005, Fig. 4a). In addition, 65.4% of genes contained at least one 5′ UTR located PAC that had an “atypical” 5′ UTR. This ratio may be even higher considering the TAIR10 annotation of 5′ UTRs has room for improvement^17^,^38^.

#### Genes containing long introns

In some cases, **n3PAS**s were concomitant with extremely long introns, or relatively long introns compared to their gene lengths. Given the fact that intron length in Arabidopsis ranged from 59 to 4162 *nt* with a median length of 100 *nt*^39^, a threshold of 800 *nt* was arbitrarily determined to categorize extreme intron length^39^. In our definition, any gene transcript isoform that contained at least one intron longer than 800 *nt*, or an intron occupied more than 1/3 of the entire gene length, was labeled as a “long intron contained” gene. There were in total 27206 protein coding genes and 2706 (9.9%) of them were labeled as “long intron contained” genes on a genome scale. Compared to the occurrence of long intron events in the genome, the frequency of “long intron” events in genes containing **n3PAS**s from the four sub-datasets was significantly higher (P-value = 0.0279, Fig. 4a).

#### Genes having a high number of introns

Another analysis of the correlation between the occurrence of **n3PAS**s and intron count also suggested the existence of positive correlation. As shown in Fig. 4b, as the number of introns and the length of a gene increased, the likelihood of detecting **n3PAS**s increased as well. In contrast, the abundance of 3′ UTR PACs remained at a constant level. Specifically, any gene with more than 30 introns was found to have at least one **n3PAS** in the PAT_LFR dataset. To further demonstrate our findings that characteristics of host genes may influence the generation of **n3PAS**s, all genes were classified into three categories: genes with only 3′ UTR poly(A) sites (Only3UTR), genes with both 3′ UTR poly(A) sites and **n3PAS**s (Both), and genes with only **n3PAS**s (OnlyNon-3UTR, which means that both PAT-Seq and DRS data utilized in this study do not provide sequence read evidence for the annotated end of 3′-UTR in TAIR10). By comparing and examining all the aforementioned characteristics among the three groups (Supplementary Figure 6), we found that genes containing at least one **n3PAS** (either Both or OnlyNon-3UTR) had a stronger correlation with aforementioned gene features (i.e., presence of spliced, diminutive or diverse beginning of 5′ UTRs, number of introns and whether introns have extreme lengths), unlike the genes in the Only3UTR group. The fact that the characterization of genes in Both and OnlyNon-3UTR groups are similar, but distinct from the genes in the Only3UTR group, re-emphasized the possibility of having different underlying regulatory mechanisms between 3′ UTR poly(A) sites and **n3PAS**s.

### Convergent genes may introduce poly(A) sites at the conjunction for both genes

**Figure 5:**
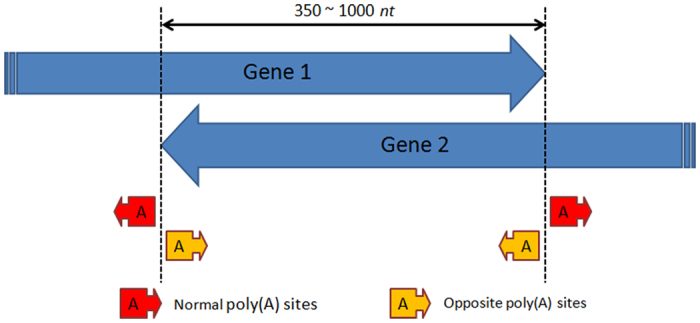
Schema of “normal” and “opposite” polyadenylation sites at the conjunction of convergently overlapping protein-coding gene regions. Theoretically, “normal” poly(A) sites are naturally present to mark the end of genes, while “opposite” poly(A) sites are presumably induced by the presence of nearby “normal” poly(A) sites from the gene on the other strand.

Arabidopsis has a very compact genome and therefore contains a multitude of overlapped gene pairs. Among them, 1402 gene pairs were overlapped convergently in opposite orientations, 138 were overlapped divergently and 226 were overlaid in the same orientations. The occurrences of different types of overlapped gene pairs implied gene endings had looser boundaries than the starting positions, especially when two endings (3′ UTR) met. Interestingly, the initial visual inspection on convergently overlapped gene pairs suggested, as exemplified in Fig. 5, that while many 3′ UTR PACs (“normal” poly(A) sites) accompany the end of genes, most genes also had a nearby PAC (“opposite” poly(A) sites) in the opposite orientation, presumably from the gene on the other strand. Also, regardless of whether “normal” 3′ UTR PACs were present or not, the probability that “opposite” PACs would be present at the genes’ ending boundaries was relatively high. Hence, an **n3PAS** was likely to occur on the other strand when the conjunction reached into the coding region of the overlapped gene. An example gene pair consisting of AT3G07590 and AT3G07600 was explicitly described in Supplementary Figure 7. To verify this phenomenon, 145 gene pairs that had an overlap region ranging from 350 to 1000 *nt* in length were selected out of 1402 convergently overlapped gene pairs. If a gene’s 3′ UTR was precisely annotated in TAIR10, then presumably a PAC in PAT_LFR would exist to support this 3′ end of the gene. Nonetheless, due to the variation of sequencing depth and the plant materials sampled for total RNA isolation in different studies, it is reasonable that some gene endings in TAIR10 were not supported by PACs in PAT_LFR. Among the 290 genes from 145 convergently overlapped gene pairs, 160 gene ending sites (“normal” PACs) were supported by PACs in PAT_LFR. Surprisingly, 123 “opposite” PACs were detected in PAT_LFR. Among these 123 PACs, 94 were annotated as 3′ UTR poly(A) sites and 29 were annotated as **n3PAS**s. The high ratio of the number of “opposite” PACs to “normal” PACs implies that convergent gene pairs are influential in enhancing the generation of polyadenylation at their overlapping regions.

### Some n3PASs are associated with Transposable Elements (TEs)

TEs are known to be capable of changing the genome structure. A previous study had determined that TEs were playing a role in the formation of polyadenylation sites in human and mouse genomes^40^, where a TE may contain the entire poly(A) site or contribute to part of it. A similar study focusing on the association of poly(A) sites and TEs in Arabidopsis was conducted in this project. The criteria of defining whether a transposable element was associated with a PAC required the distance between them to be less than 40 *nt*^40^. A total of 31189 TEs were annotated in TAIR10. Using the criteria of requiring a minimum distance of 40 *nt* between TE and PAC to define the association between them^40^, 1591 sense genic PACs were detected as associated with TEs, consisting of 383 **n3PAS**s and 1208 3′ UTR PACs, which were potentially derived from their associated TEs. The number of PAC associated TE families followed the order of Helitron > DNA > LINE > SINE (Supplementary Table 1). Unlike the animal study where retrotransposon was the major type of PAC associated TEs^40^, the DNA transposon was the major type in Arabidopsis. It seems that PACs associated with TEs do not show any obvious preference among different types of TEs.

## Discussion

Polyadenylation is known to mark the 3′ boundary of genes. The majority of APAs occur in 3′ UTR regions, and they do not alter the coding sequences of the full length genes. However, if APAs happen in genic regions upstream of the 3′ UTR, *i.e.*, **n3PAS**s, the encoded sequence length might be truncated, thus affecting resultant protein products. Along with the result from our study, increasing evidence has supported the authenticity and functionality of those **n3PAS**s^3^ and the differences between **n3PAS**s and 3′ UTR poly(A) sites in terms of regulatory mechanisms^20^. This poses the question of what biological relevance and regulatory mechanisms are behind the creation of **n3PAS**s. In our study, we notice that the frequency of a non-3UTR polyadenylation event is positively correlated with specific genic features (i.e., presence of spliced, diminutive or diverse beginning of 5′ UTRs, number of introns and whether introns have extreme lengths) and hence hypothesize that these aforementioned genic features potentially contribute to the generation of **n3PAS**s.

Compared with 3′ UTR located PACs, the average PAT read abundance for **n3PAS**s is relatively low. Although non-3UTR PAT reads account for no more than 3% of all sense PAT reads, the total number of non-3UTR PACs could be as high as 11782. Thus, it’s not surprising **n3PAS**s have long been considered to be sequencing artifacts. Even in our study, we have to acknowledge that the total number of PACs is arguably correct, as the controversy of the presence of cdsExonPASs and only a limited number of **n3PAS**s have literally been validated by wet-lab testing^3^. Depending on the specific region where PACs are located, **n3PAS**s are classified into three categories/groups: 5′ UTR PACs (5UtrPASs), CDS intronic PACs (cdsIntronPASs) and CDS exonic PACs (cdsExonPASs). Firstly, 5UtrPASs have been detected occasionally by other researchers^3^,^12^,^36^. The 5′ UTR of the mRNA is critical to the initiation and efficiency of translation, and the “atypical” 5′ UTR has been documented to play a role in regulating its transcriptional and translational activity to a different extent. For instance, many more 5′ UTRs (16.5%) contain introns than 3′ UTRs (3%), as the asymmetry could be attributed to nonsense mediated decay of mRNA^41^. Introns in the 5′ UTR are capable to regulate the expression of its host gene^42^. Also, genes might be partially silenced when polyadenylation occurs in the 5′ UTR. Agreed by the functionality analysis, a good portion of genes with 5UtrPASs are associated with stress regulation, which leads us to suspect that the appearance of 5UtrPASs might be related with an unidentified regulatory mechanism for genes to terminate transcription on purpose, or just the evidence of hidden gene units. More than two thirds of genes contain at least one 5′ UTR located PAC having an “atypical” 5′ UTR. A closer inspection of the remaining 34.6% (207) of PACs suggests these PACs were likely correlated with other possible influential features, including nearby small RNA units and TEs. Secondly, regarding cdsIntronPASs, both PAT-Seq and DRS based data suggest a very similar distribution of cdsIntronPASs, and the association between splicing and cdsIntronPASs seems to be valid because some cdsIntronPASs are known to be suppressed by U1 snRNP^43^ and cdsIntronPASs are normally located in longer introns. Moreover, the intron before the last exon is likely to contain a cdsIntronPAS, which is consistent with a previous study as well^34^. We claimed that longer introns were associated with **n3PAS**s, but meanwhile, an unavoidable fact is that a longer gene, or a gene with long introns, may embrace a higher chance to form **n3PAS** sites. To test this hypothesis, we examined the connection between gene length and **n3PAS**s (Supplemental Figure S8). Interestingly, such comparison of the gene length between genes with and without **n3PAS** did not show significant difference (P-value = 0.624). Therefore, this suggests that the impact of longer introns on **n3PAS** occurrence is still true. Lastly, cdsExonPASs have also been detected in previous studies^12^,^34^ and these sites are significantly more abundant in the PAT-Seq datasets compared to the DRS based dataset by using the same bioinformatics approach. Their authenticity is still debatable, though some cdsExonPASs have been verified by 3′ RACE^3^. However, it is suggested that they might actually be artifacts caused by internal priming on A-rich sequence stretches, or by other possible reasons during cDNA library preparation and the sequencing itself^12^. This discrepancy of cdsExonPASs between two different sequencing techniques raises an interesting question of how to select the best sequencing approach for polyadenylation detection in plants. These two approaches share very similar performance (e.g., overall genome-wide PAS profiles) despite the discrepancy for this subset of **n3PAS**s (i.e, cdsExonPASs). In our view, the selection between PAT-Seq versus DRS relies largely on the availability and budgets/expenses to the individual labs. Notably, the abundance of cdsExonPASs in PAT_F is smaller comparing to other PAT-L and PAT-R datasets, indicating different plant organs may display a varied PAC distribution.

Overlapped genes have been reported to exhibit interesting interactions between themselves^44^,^45^,^46^. Here, we firstly propose that “opposite” poly(A) sites are generated at the conjunction of convergently overlapped gene pairs. In other words, polyadenylation on one strand would enhance polyadenylation site formation in the gene on the other strand. Among the overlapped genes identified in our study, 123 “opposite” poly(A) sites were found in 290 genes, exemplified in Fig. 5. This is a high proportion for “opposite” poly(A) sites when considering only 160 “normal” poly(A) sites were detected from 290 ending sites of genes in PAT_LFR, where 130 were not detected with “normal” 3′ UTR poly(A) sites from our data. In all, the high occurrence of “opposite” poly(A) sites suggests their presence is not a coincidence and is indicative of a potential hidden mechanism for polyadenylation. The hypothetic mechanism we propose here is that the pause of polyadenylation machinery on one strand can facilitate the recruitment of polyadenylation auxiliary factors on the other strand, thus empowering the formation of “opposite” poly(A) sites. Not all identified convergent conjunctions contained “opposite” poly(A) sites. This can be explained by the fact that an appropriate poly(A) signal is required even if sufficient auxiliary poly(A) factors are present. Additionally, the variation of sequencing methods and plant tissue materials sampled among different datasets could affect the detection of these sites. Meanwhile, the “opposite” poly(A) sites detected could be antisense poly(A) sites as well, but many examples from TAIR10 verify that these “opposite” poly(A) sites are actually from a shorter sense transcript instead of an antisense transcript, such as the one shown in Supplementary Figure 7. However, this cannot be fully decided until full length sequencing is performed.

Accounting for at least of 10% of the genome, TEs in *Arabidopsis* play an important role in shaping the genome structure, as they do in many other organisms through evolution^47^. Regarding the association between TEs and poly(A) sites, a previous bioinformatics study on human and mouse have determined 3′ UTR located poly(A) sites are more conserved than their upstream **n3PAS**s^40^. Non-conserved poly(A) sites (mostly **n3PAS**s) are associated with TEs to a much higher degree than conserved ones (mostly 3′ UTR poly(A) sites)^40^. Similar results have been also discovered in our study in which hundreds of PACs are associated with TEs. As indicated from our study, by changing the surrounding sequence context or directly generating an appropriate poly(A) site, the movement of TEs may produce both 3′ UTR and novel **n3PAS**s along the genes in Arabidopsis.

In conclusion, the investigation of genome-wide **n3PAS**s from a comprehensive dataset in this study provided a new perspective and deeper insight into the scope of polyadenylation mechanism study in Arabidopsis. The occurrence of **n3PAS**s were demonstrated to be positively correlated with specific genetic features such as “atypical” 5′ UTRs, a high quantity of introns, long introns, overlapping gene pairs and nearby TEs. Our analyses revealed an additional polyadenylation mechanism that augments current understanding, namely, the surrounding genetic configuration of host genes could also influence the generation of polyadenylation sites. In the future, extensive manual curation and wet-lab validation is needed to better explain the processing mechanism of alternative polyadenylation, and the study of **n3PAS**s should not be limited only to the plant study.

## Additional Information

**How to cite this article**: Guo, C. *et al*. A Genome-wide Study of "Non-3UTR" Polyadenylation Sites in *Arabidopsis thaliana*. *Sci. Rep.* **6**, 28060; doi: 10.1038/srep28060 (2016).

## Supplementary Material

Supplementary Information

## Figures and Tables

**Table 1:** Genomic distribution of PATs/PACs in the datasets.

Dataset	Sense PATs^a^/PACs^b^				Intergenic PATs^a^/PACs^b^	Antisense PATs^a^/PACs^b^
	*5UtrPASs*	*cdsExonPASs*	*cdsIntronPASs*	*3UtrPASs*		
PAT_L	93586/990	121148/5249	69859/2586	4822975/20878	960450/16280	499806/5130
PAT_F	18053/414	12085/573	66571/1426	9325848/21478	274947/9139	1056046/4469
PAT_R	180256/1618	104338/6713	176293/5406	18478178/24127	2191611/24170	3474717/8591
PAT_LFR^c^	263588/744	169287/7330	262791/3708	31585245/25202	1677324/11427	3059319/6675
DRS_L	85027/825	20741/612	205174/3057	10417997/19969	818065/18839	1488450/5094

^a^The total number of PATs: poly(A) tag reads that support high-quality individual poly(A) sites(i.e., ≥3 PAT reads).

^b^The total number of poly(A) site clusters (PACs) in the respective genomic regions.

^c^PAT_LFR is the dataset combining PAT_L, PAT_F and PAT_R together.
