# Supplementary material for: A Genome-wide Study of “Non-3UTR” Polyadenylation Sites in Arabidopsis thaliana
Source: Sci Rep. 2016 Jun 15;6:28060. doi: 10.1038/srep28060 (PMC4908657; doi:10.1038/srep28060)
Supplement: Supplementary Information [file srep28060-s1.pdf]

Supplementary Information for:

A Genome-wide Study of "Non-3UTR" Polyadenylation Sites in *Arabidopsis thaliana*

Cheng Guo[1], Matthew Spinelli[1], Man Liu[1], Qingshun Q. Li[1,2,3*] and Chun Liang[1*]

[1] Department of Biology, Miami University, Oxford, OH 45056, USA

[2] Key Laboratory of the Ministry of Education for Costal and Wetland Ecosystems, College of the Environment and Ecology, Xiamen University, Xiamen, Fujian 361102, China

[3] Graduate College of Biomedical Sciences, Western University of Health Sciences, Pomona, CA 91766, USA

*To whom correspondence should be addressed: liangc@miamioh.edu and qqli@westernu.edu

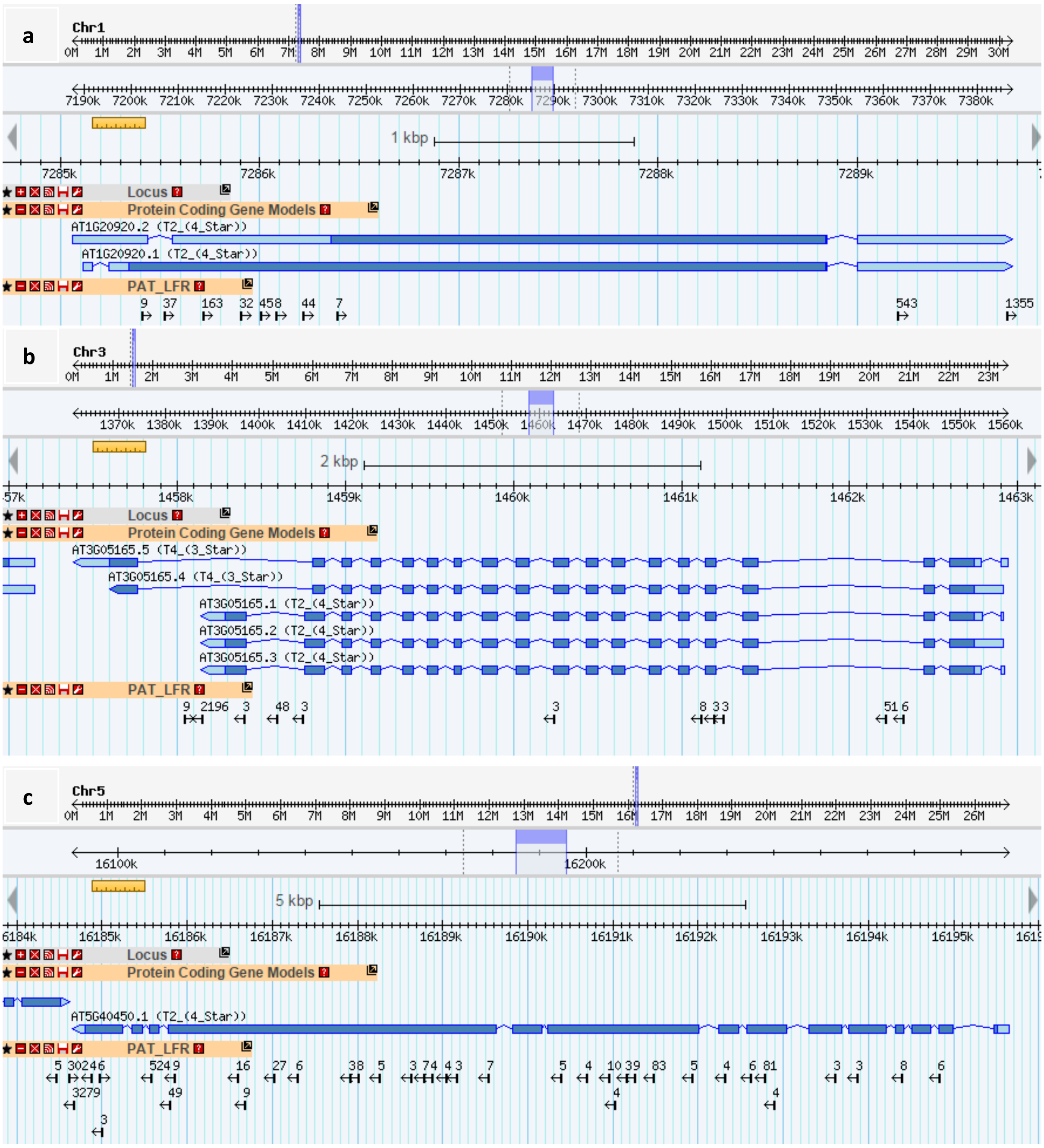

Figure S1. Examples of n3PASs in different genic regions. The black arrows and numbers at the bottom panel indicate the position, direction and abundance of supporting PAT reads of the poly(A) site clusters in dataset PAT_LFR.
(a) 5UtrPASs in gene AT1G20920. The model of the longer transcript AT1G20290.2 is used for annotating the region containing PACs. The gene also contains cdsExonPASs and 3' UTR poly(A) sites as well. (b) cdsIntronPAS in gene AT3G05165.The longest transcript AT3G05165.5 is used for annotating the region containing PACs. The gene also contains cdsExonPASs and 3' UTR poly(A) sites as well. (c) cdsIntronPAS in gene AT5G40450. The gene also contains 3' UTR poly(A) sites as well.

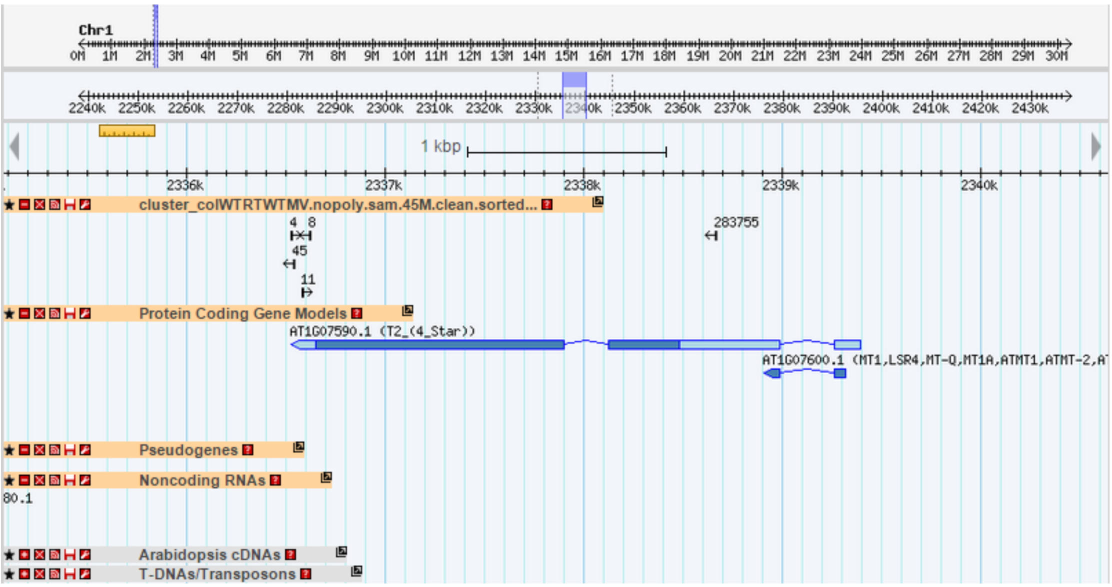

Figure S2. Snapshot of poly(A) sites around the gene AT1G07590 and AT1G07600. A highly expressed PAC site with 283755 PAT reads is found in the 5' UTR of AT1G07590. and the downstream region of gene AT1G07600. Instead of annotating this as a 5UtrPAS for AT1G07590, the TAIR10 actually annotates it as the evidence of a relative short gene AT1G07600. In sum, we suspect this PAC (PATs=283755) confuses the annotation of TAIR10 for the annotation of gene AT1G07600.

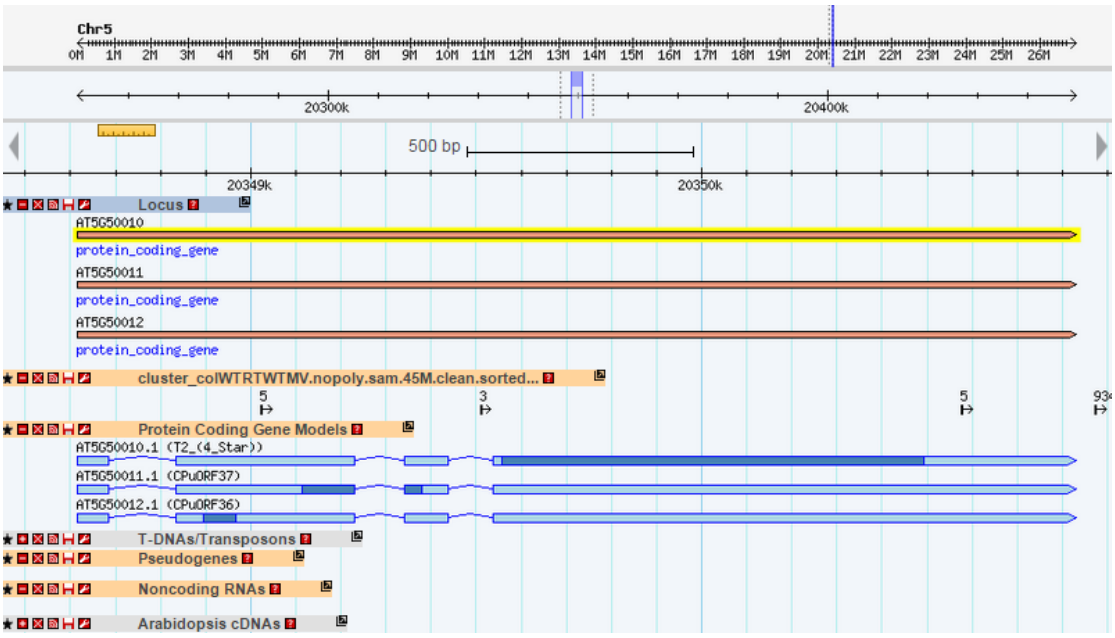

Figure S3. Snapshot of n3UTR and 3' UTR poly(A) sites in gene cluster of AT5G50010, AT5G50011 and AT5G50012.

Although sharing the same sequence, the three genes in this gene cluster have different 5' UTRs, coding region and 3' UTR annotations, which seem to be problematic. Noticing that a poly(A) site (PATs=5, left) is just located in the downstream of CDS of AT5G50012.1, a poly(A) site (PATs=3) is just located in the downstream of CDS of AT5G50011.1 and another poly(A) site (PATs=5, right) is just located in the downstream of CDS of AT5G50010.1, it is suspected that these poly(A) sites are the reason causing the annotation problem in TAIR10 for this gene cluster.

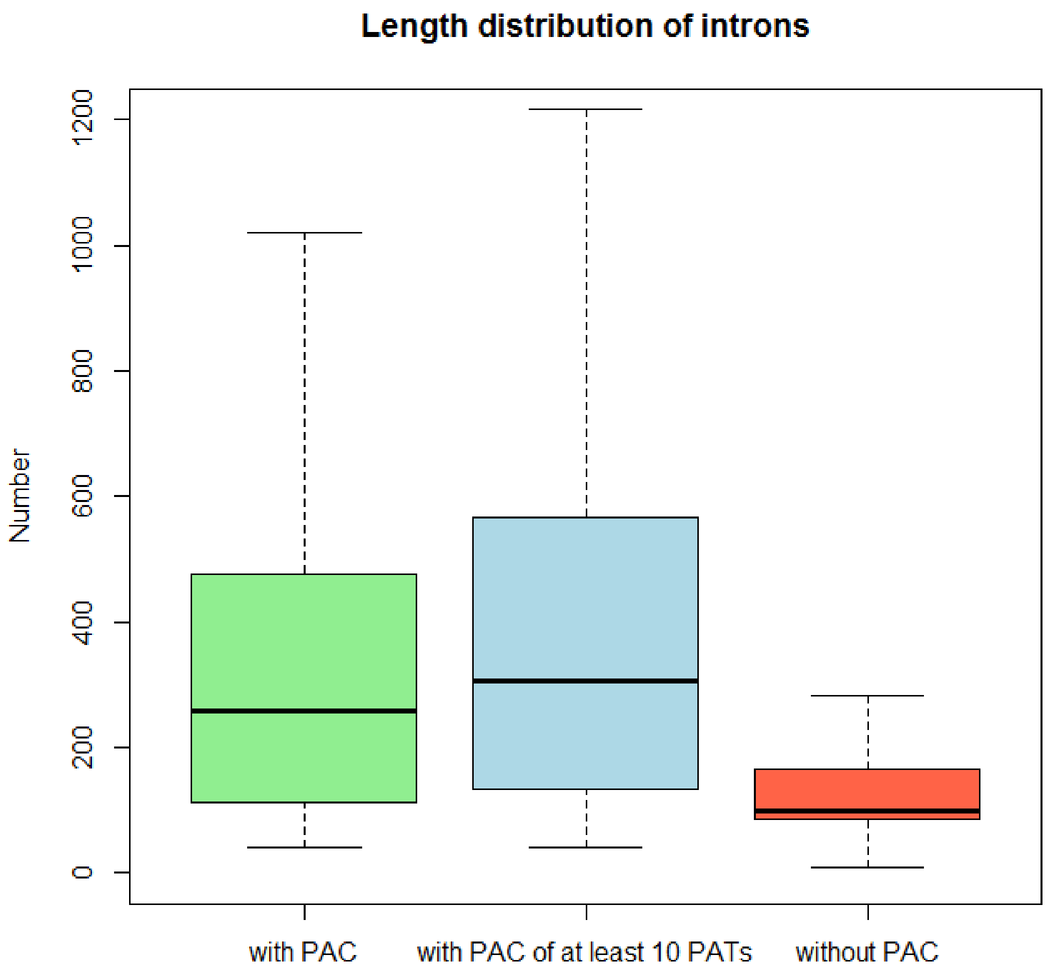

Figure S4. Boxplot of the length distributions of introns among different intron groups. The intron groups include intron group with detected PACs, intron group with detected PACs, who have at least 10 PATs, and intron group without any detected PACs.

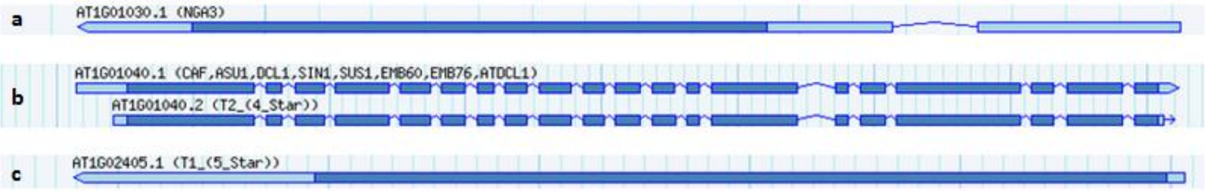

Figure S5. Examples of “atypical 5’ UTR” events in genes.
(a) The 5’ UTR in gene AT1G01030 is defined as a “spliced 5’ UTR” apparently because of the splicing. (b) Gene AT1G01040 has two annotated transcripts where the 5’ UTR of the transcripts do not have a consistent starting site (in other cases not shown, the ending sites are inconsistent). Therefore the 5’ UTR is defined as a “ambiguous 5’ UTR”. (c) The 5’ UTR in gene AT1G02450 is only 7 bp in length, and is classified into the “extreme short 5’ UTR” group.

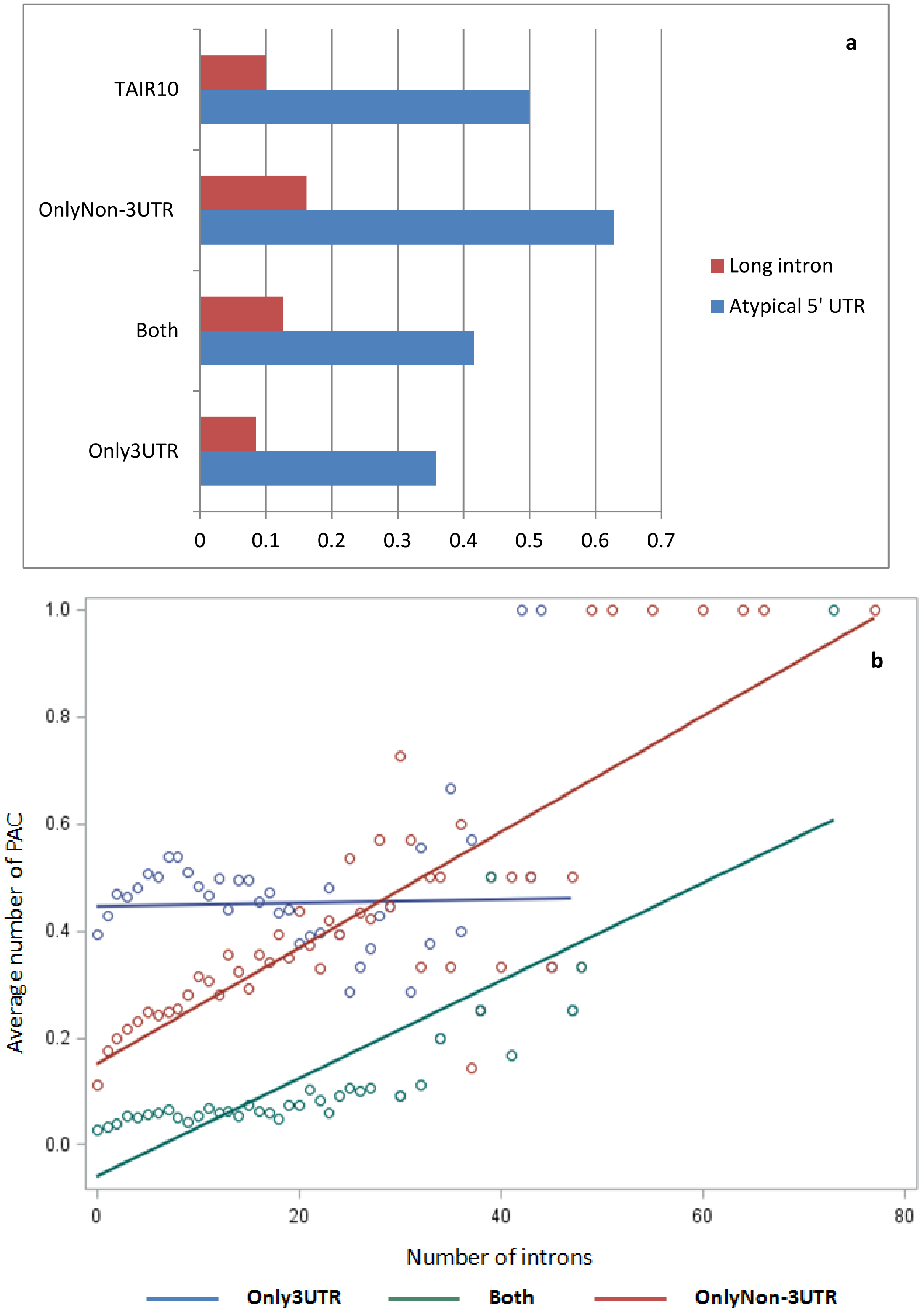

Figure S6. The genome-wide comparison of gene characteristics among genes with only 3' UTR poly(A) sites (Only3UTR), genes with both 3' UTR and n3PASs (Both), genes with only n3PASs (OnlyNon-3UTR).

(a) The comparison of the ratio of atypical 5' UTR and long intron among groups. (b) The comparison of average abundance of PACs with respective intron numbers among groups.

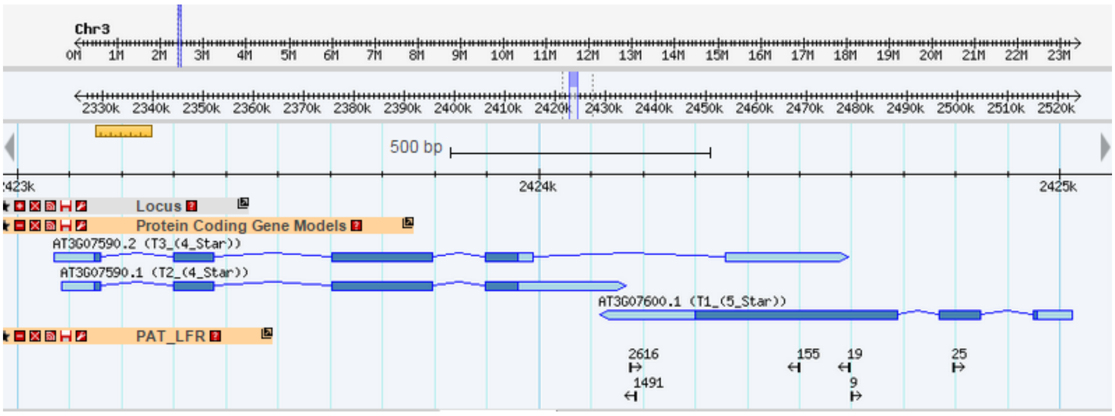

Figure S7. The snapshot of convergent gene pairs, AT3G07590 and AT3G07600.
As illustrated, AT3G07590 contains two transcripts and the longer one convergently overlaps with gene AT3G07600 by around 500 *nt*. The right/left normal poly(A) sites (supported by 9 and 1491 PATs) of the gene pair are detected in dataset PAT_LFR. Additionally, both right/left opposite poly(A) sites (supported by 2616 and 19 PATs) are also detected. Also, the shorter transcript AT3G07590 verifies that the left opposite poly(A) site is not an antisense poly(A) transcript, but a sense transcript with a poly(A) tail derived from gene AT3G07590.

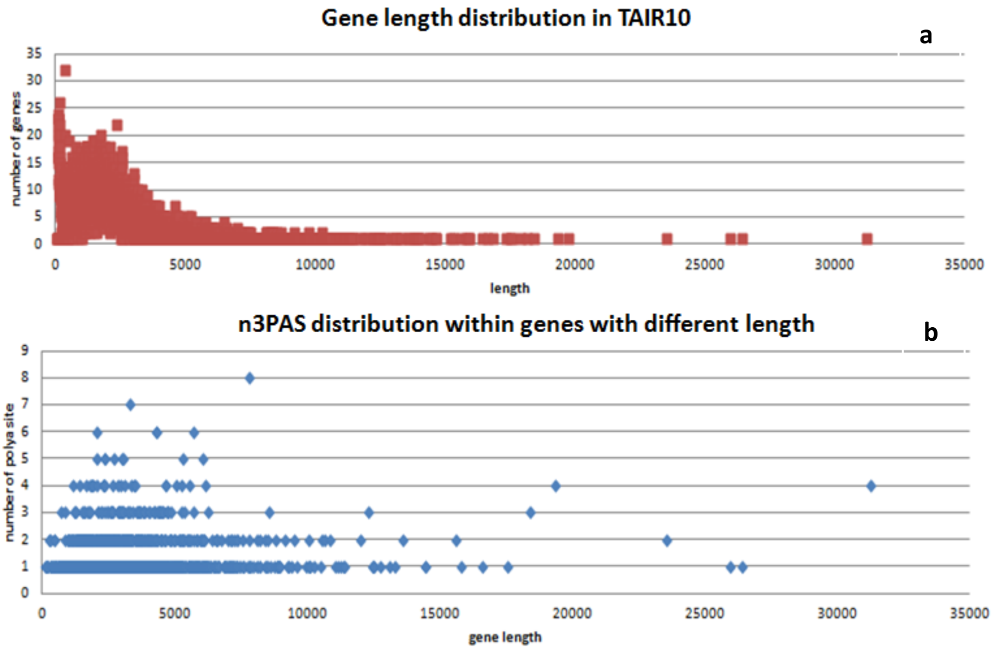

Figure S8. The comparison of the length distribution of gene in TAIR10 and the n3PASs distribution of the genes with n3PAS.

(a). The gene length distribution in TAIR10. X axis represents the length of gene, Y axis represents the number of genes with such length. (b). the n3PAS distribution within the n3PASs contained genes. X axis represents the length of gene, Y axis represents the total number of n3PASs detected in such genes. We speculate the occurrence of n3PAS is not directly correlated with the length of gene. Also, a follow up t-test comparing the gene length between genes with and without n3PAS did not show significant difference (P-value = 0.624).

Table S1. The abundance of PACs associated transposable elements in Arabidopsis

TE family	Number
DNA	63
DNA/En-Spm	50
DNA/Harbinger	30
DNA/HAT	63
DNA/Mariner	11
DNA/MuDR	329
DNA/Pogo	21
DNA/Tc1	6
LINE/L1	67
LINE	6
LTR/Copia	92
LTR/Gypsy	44
RathE1_cons	14
RathE2_cons	1
RathE3_cons	3
RC/Helitron	776
SINE	8
Unassigned	7
